# Drug-Induced Reorganisation of Lipid Metabolism Limits the Therapeutic Efficacy of Ponatinib in Glioma Stem Cells

**DOI:** 10.3390/pharmaceutics16060728

**Published:** 2024-05-29

**Authors:** Paula Aldaz, Ana Olias-Arjona, Irene Lasheras-Otero, Karina Ausin, Marta Redondo-Muñoz, Claudia Wellbrock, Enrique Santamaria, Joaquin Fernandez-Irigoyen, Imanol Arozarena

**Affiliations:** 1 Cancer Signaling Unit, Navarrabiomed, Hospital Universitario de Navarra (HUN), Universidad Pública de Navarra (UPNA), Irunlarrea 3, 31008 Pamplona, Spain; ana.olias.arjona@navarra.es (A.O.-A.); ilasheras@viralgenvc.com (I.L.-O.); marta.redondo.munoz@navarra.es (M.R.-M.); claudia.wellbrock@unavarra.es (C.W.); 2 Health Research Institute of Navarre (IdiSNA), 31008 Pamplona, Spain; karina.ausin.perez@navarra.es (K.A.); enrique.santamaria.martinez@navarra.es (E.S.); joaquin.fernandez.irigoyen@navarra.es (J.F.-I.); 3 Proteomics Platform, Navarrabiomed, Hospital Universitario de Navarra (HUN), Universidad Pública de Navarra (UPNA), 31008 Pamplona, Spain; 4 Department of Health Sciences, Universidad Pública de Navarra (UPNA), 31008 Pamplona, Spain; 5 Clinical Neuroproteomics Unit, Navarrabiomed, Hospital Universitario de Navarra (HUN), Universidad Pública de Navarra (UPNA), 31008 Pamplona, Spain

**Keywords:** glioblastoma, glioma stem cell, ponatinib, cholesterol, sphingolipids, lipid metabolism

## Abstract

The standard of care for glioblastoma (GBM) involves surgery followed by adjuvant radio- and chemotherapy, but often within months, patients relapse, and this has been linked to glioma stem cells (GSCs), self-renewing cells with increased therapy resistance. The identification of the epidermal growth factor receptor (EGFR) and platelet-derived growth factor receptor (PDGFR) as key players in gliomagenesis inspired the development of inhibitors targeting these tyrosine kinases (TKIs). However, results from clinical trials testing TKIs have been disappointing, and while the role of GSCs in conventional therapy resistance has been extensively studied, less is known about resistance of GSCs to TKIs. In this study, we have used compartmentalised proteomics to analyse the adaptive response of GSCs to ponatinib, a TKI with activity against PDGFR. The analysis of differentially expressed proteins revealed that GSCs respond to ponatinib by broadly rewiring lipid metabolism, involving fatty acid beta-oxidation, cholesterol synthesis, and sphingolipid degradation. Inhibiting each of these metabolic pathways overcame ponatinib adaptation of GSCs, but interrogation of patient data revealed sphingolipid degradation as the most relevant pathway in GBM. Our data highlight that targeting lipid metabolism, and particularly sphingolipid degradation in combinatorial therapies, could improve the outcome of TKI therapies using ponatinib in GBM.

## 1. Introduction

Glioblastoma (GBM; for acronyms, see Supplementary Table S1) remains among the cancers with poorest prognosis with a median overall survival in glioblastoma patients of only 15 months after diagnosis; this has not changed for almost 20 years [1]. After surgery, most patients are treated with radiotherapy and the chemotherapeutic agent temozolomide, followed by temozolomide as a single agent [2]. However, adjuvant radio- and chemotherapies barely extend GBM patients’ survival; most often tumours regrow and recurrent tumours do not respond to alternative treatments. Over the last two decades, mutations, amplifications, or deletions of genes such as *IDH1*, *NF1*, *PTEN*, *PDGFRA* or *EGFR* have been identified [3,4,5] and our knowledge of the molecular events leading to gliomagenesis has improved significantly. Furthermore, genome-wide analyses of large patient cohorts have identified clinically relevant GBM subtypes such as classical, proneural, or mesenchymal, which correlate with particular cellular states and tumour microenvironments, and have prognostic implications [3,4,5,6,7]. Functional analyses of PDGFRA and EGFR have shown that these receptor tyrosine kinases (RTKs) act as drivers for GBM cell growth, and mice genetically engineered to express deregulated PDGFR or EGFR signalling in an adequate genetic background, develop gliomas [8,9]. This pre-clinical and clinical evidence in GBM patients led to the assessment of small-molecule tyrosine kinase inhibitors (TKIs) targeting RTKs in numerous clinical trials [10].

Unfortunately, small cohort sizes and great heterogeneity in disease and prior therapy, as well as restrictions for some drugs in crossing the blood–brain barrier are limiting the conclusions derived from RTK targeting trials [1]. Moreover, the majority of patients in recent TKI trials had recurrent GBM and had not been stratified for tumour-specific expression of potential drug targets. Apart from all these challenges, insufficient knowledge of GBM-specific resistance mechanisms to TKIs hampers the clinical implementation of these targeted therapies in GBM patients [1].

Adaptation to drug insult leading to resistance is a significant challenge to targeted therapies in all cancer types. In GBM, therapeutic failures and recurrences after radio- and chemotherapy are thought to originate from a subpopulation of stem-like glioblastoma stem cells (GSCs), undifferentiated tumour cells with self-renewal capacity and increased resistance to therapy [11]. While the role of GSCs in the context of conventional radio- and chemotherapy has been extensively studied, little is known about the adaptive response of GSCs to TKIs. Building on our recent study on PDGFR activation in the context of glucocorticoid signalling in GBM [12], we sought to better understand the response of GSCs to ponatinib (Iclusig^®^), a multi-target TKI used for the treatment of chronic myelogenous leukaemia (CML). Apart from BCR-ABL, it has high affinity for PDGFR, and most importantly, has demonstrated promising blood–brain barrier (BBB) penetrance in mice [13] and activity in acute lymphocytic leukaemia (ALL) patients with central nervous system (CNS) relapse [14].

## 2. Materials and Methods

### 2.1. Cell Culture and Reagents

T98G (CRL-1690) glioma cell line was purchased from ATCC (American Type Culture Collection) and GSC-11 and GSC-23 glioma stem cells derived from patients were a kind gift from Dr Marta Alonso (Solid Tumours and Biomarkers, Foundation for the Applied Medical Research, Pamplona, Spain). In adhesion conditions, the T98G cell line was cultured in Dulbecco’s Modified Eagle’s Medium (DMEM) (cat#11995065, Gibco, Waltham, MA, USA), supplemented with 10% foetal bovine serum (FBS) (cat# 10500064, Gibco). GSC-11 and GSC-23 cells were continuously cultured in suspension culture using NSC medium consisting of DMEM/F-12 (Sigma, Madrid, Spain) supplemented with N2, B27 supplements (Thermo Fisher, Waltham, MA, USA), and basic fibroblast growth factor (20 ng/mL), and epidermal growth factor, (20 ng/mL) (Sigma). Cells were maintained in a humidified atmosphere of 5% CO_2_ at 37 °C. Ponatinib (PONA) was from Selleckchem (cat#S1490; Newmarket, UK). Ranolazine (HY-17401), Simvastatin (HY-17502), and Lovastatin (HY-N0504) were from MedchemExpress (Monmouth Junction, NJ, USA). Decanoic acid (W236403), ceranib-2 (SML0607) and Thioridazine (T9025) were from Sigma/Merck (Madrid, Spain). All drugs were reconstituted in DMSO.

### 2.2. Neurosphere Formation and Self-Renewal Analysis

For neurosphere assays, T98G cells were transferred from adhesion conditions to suspension using NSC medium. To allow neurosphere formation, T89G, GSC-11 and GSC-23 were cultured in NSC medium in non-coated 12-well flat-bottom plates for 7 days, after which they were disaggregated mechanically and enzymatically with accutase (Gibco) and seeded again to form secondary (2ry) neurospheres for another 7 days in order to study self-renewal capacity. For quantification studies, 0.5 × 104 disaggregated GSC-11 or GSC-23 cells or 0.2 × 104 disaggregated T98G cells per well were seeded and after 24 h the respective reagents (decanoic acid or inhibitors) at the indicated concentrations were added. Analysis was performed after 7 days by determining the number of newly formed spheres using a light microscope.

### 2.3. Subcellular Fractionation of Neurosphere Cells

Ponatinib (200 nM)-treated T98G neurosphere cells were centrifuged, the cell pellets washed with cold PBS. A total of 500 μL of NP40 (1%) buffer with protease inhibitors was added and lysed cells were harvested. After centrifugation (1 min, 13,000 rpm) at 4 °C, the supernatant was collected in a fresh Eppendorf tube (cytosolic fraction). The pellet was washed with PBS and centrifuged for 1 min (13,000 rpm, 4 °C), and the supernatant was discarded. The pellet (peri/nuclear fraction) was resuspended in 50 μL of lysis buffer (7M urea, 2M thiourea, 50 mM DTT) and left on ice for 30 min, with vortexing every 10 min. After a sonication step, the lysate was centrifuged for 20 min at 20,000× *g* for 15 °C. The supernatant was transferred to a new Eppendorf tube and the pellet discarded. Protein concentration of both subcellular fractions was measured with the Bradford assay kit (Bio-Rad, Philadelphia, PA, USA).

### 2.4. Mass Spectrometry

#### 2.4.1. MS/MS Spectral Library Generation

For quantitative proteomics, the sequential window acquisition of all theoretical mass spectra (SWATH) method was used. A SWATH-MS assay library was generated using two independent pools of the C and PN fractions. For protein digestion, 20 μg of total protein in each sample was reduced (10 mM DTT, 30 min, RT) and alkylated (30 mM iodoacetamide, 30 min RT), trypsin (Promega, Madison, WI, USA) was added (enzyme:protein, 1:50 *w*/*w*) and the samples were incubated at 37 °C for 16 hours. Digestion was quenched by acidification (pH < 6) with acetic acid. After protein digestion, peptide cleaning was performed using an Oasis PRiME MCX 96-well µElution Plate (Waters Ltd., Wilmslow, UK), which was dried in a SpeedVac and reconstituted with 120 µL of 5 mM ammonium bicarbonate pH 9.8, and injected into an ÄKTA pure 25 system (GE Healthcare Life Sciences, Chicago, IL, USA) with a high pH stable X-Terra RP18 column (C18; 1 mm × 150 mm; 5 μm) (Waters Ltd.). Peptides were eluted with a mobile phase (B: 5mM ammonium bicarbonate in 90% ACN at pH 9.8) of 30–70% linear gradient over 45 min (A: 5 mM ammonium bicarbonate in water at pH 9.8; B: 5 mM ammonium bicarbonate in 90% ACN at pH 9.8). The UV signal was monitored at 215 nm, 17 fractions were collected and evaporated in a vacuum. Purification and concentration of peptides was performed using C18 Zip Tip Solid Phase Extraction (Millipore, Burlington, MA, USA) and reconstituted in 2% ACN, 0.5% FA prior to mass spectrometry analysis. MS/MS datasets for spectral library generation were acquired on a TripleTOF 5600+ mass spectrometer (Sciex, Framingham, MA, USA) interfaced to the Eksigent nanoLC ultra 2D pump system (Sciex). MS/MS data acquisition was performed using AnalystTF 1.7 (Sciex), and spectra files were processed through ProteinPilot Software (v.5.0, Sciex) using ParagonTM Algorithm (v.4.0.0.0) for database search [15], and Progroup™ Algorithm for protein grouping. The false discovery rate (FDR) was determined using a non-linear fitting method [16] and results considered were those reporting a 1% Global FDR or better).

#### 2.4.2. Quantitative Proteomics by SWATH Analysis

Protein extracts (20 µg) from each sample (n = 48) were subjected to in-solution digestion as described above. For SWATH-MS-based experiments, the TripleTOF 5600+ instrument was configured as described by Gillet et al. [17]. Using an isolation width of 16 Da, a set of 37 overlapping windows was constructed covering the mass range 450–1000 Da. A total of 1 μg of each sample was loaded onto an Acclaim™ PepMap™ 100 C18 trap column (0.1 × 20 mm, particle size 5 µm; ThermoFisher, Scientific, Waltham, MA, USA) and desalted with 100% water 0.1% formic acid at 2 μL/min for 10 min. The peptides were loaded onto an Acclaim™ PepMap™ 100 C18 column (0.075 × 250 mm, particle size 3 µm; ThermoFisher) equilibrated in 2% acetonitrile 0.1% FA. Peptide elution was carried out with a linear gradient of 2 to 40% B in 120 min (mobile phases A: 100% water 0.1% formic acid (FA) and B: 100% acetonitrile 0.1% FA) at a flow rate of 300 nL/min. Eluted peptides were infused in the mass spectrometer. The Triple TOF was operated in SWATH mode, in which a 0.050 s TOF MS scan from 350 to 1250 *m*/*z* was performed, followed by 0.080 s product ion scans from 230 to 1800 *m*/*z* on the 37 defined windows (3.05 s/cycle). The collision energy was set to optimum energy for a 2+ ion at the centre of each SWATH block with a 15 eV collision energy spread. The resulting ProteinPilot group file from library generation was loaded into PeakView^®^ (v2.1, Sciex), and peaks from SWATH runs were extracted with a peptide confidence threshold of 99% (Unused Score ≥ 1.3) and FDR lower than 1%. For this, the MS/MS spectra of the assigned peptides were extracted with ProteinPilot, and only the proteins that fulfilled the following criteria were validated: peptide mass tolerance lower than 10 ppm, 99% confidence level in peptide identification, and complete b/y ions series found in the MS/MS spectrum. Only proteins quantified with at least two unique peptides were considered. The quantitative data obtained by PeakView^®^ were analysed using Perseus software (version 1.6.14.0) [18] for statistical analysis and data visualisation.

### 2.5. Western Blot Analysis

Immunoblots were performed following standard procedures [19]. Primary antibodies were the following: p-PDGFβ (Y740) (cat# 3168; Cell Signaling, Danvers, MA, USA), H3 (ab1791; Abcam (Cambridge, UK)) and GAPDH (CB1001) and β-Actin (cat# A5441) from Sigma/Merck. Detection was through enhanced chemiluminescence ECL using Horse Radish Peroxidase (HRP)-coupled secondary antibodies (GE Healthcare) and NOVEX ECL Chemi Substrate (ThermoFisher).

### 2.6. ASAH1 Overexpression

T98G cells were transfected with pcDNA5/TO-ASAH1 [20] using Lipofectamine and were selected with hygromycin B (200 μg/mL) (Invivogen, San Diego, CA, USA) to generate T98G ASAH1 cells.

### 2.7. Data Analysis and Statistics

The proteome-based interactome was analysed using STRING [21]. To minimise false positives as well as false negatives, all interactions tagged as “low-confidence” (<0.4) in STRING database were eliminated. To elucidate the biological impact of the proteostatic impairment induced by ponatinib, we performed a biological enrichment analysis in Metascape [22] using default settings (minimum overlap: 3; minimum enrichment: 1.5; *p* < 0.01). For bar graph analyses, one-way ANOVA or Student’s *t* test in GraphPad Prism version 9.00 (GraphPad Software, San Diego, CA, USA) were used, and data represent results from at least 3 replicates. Expression and survival data were extracted from cbioportal [23,24] analysing the 2008 and 2013 TCGA patient cohorts [3,4] and further analysed in GraphPad Prism; log-rank test was used for Kaplan–Meier survival analyses. For the relative expression score, the average expression of genes identified in the respective heatmaps was considered.

## 3. Results

### 3.1. Ponatinib Induces Changes in Subcellular Protein Localisation in T98G Neurospheres

**Figure 1:**
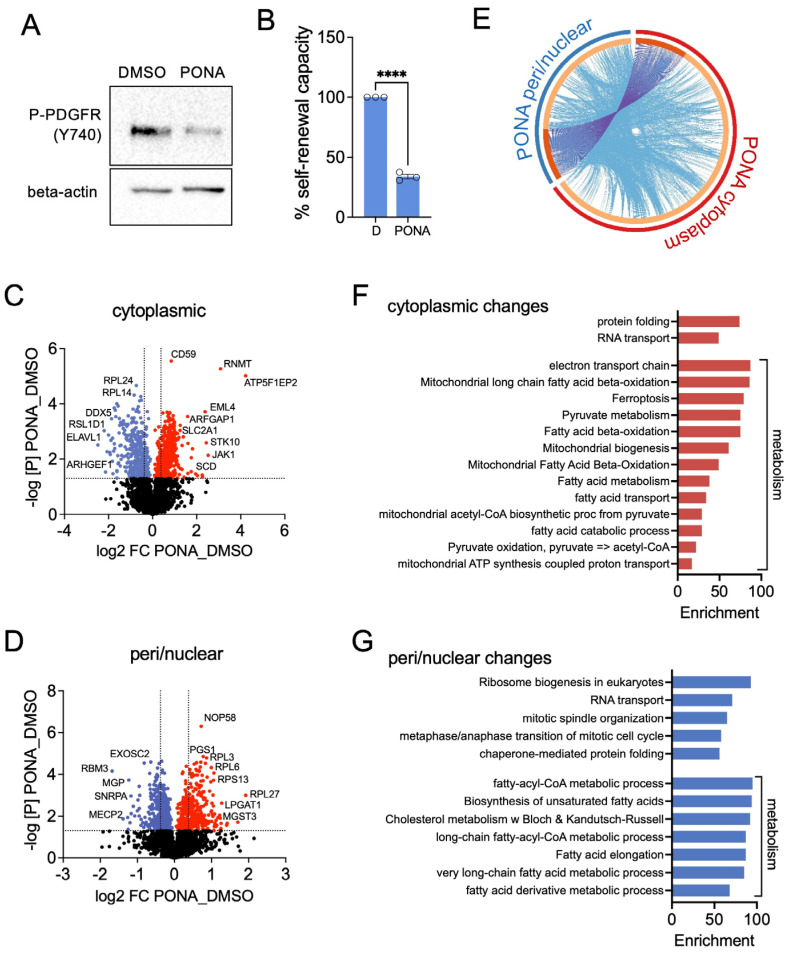
Subcellular proteome modulation induced by ponatinib. (**A**) Western blot analysis of T98G cells treated with DMSO or ponatinib for phospho-PDGFR. Beta-actin serves as loading control; (**B**) self-renewal analysis of T98G cells treated with DMSO (D) or ponatinib. Data represent mean ± SEM. **** *p* ≤ 0.0001; (**C**,**D**) volcano plots for fold change (FC) in protein expression in response to ponatinib in (**C**) the cytosolic and (**D**) the peri/nuclear fraction. The expression of significantly up-regulated or down-regulated proteins is represented in red and blue, the dotted lines indicate FC > 1.3; *p* < 0.05; (**E**) circos plot for differentially expressed proteins. Dark orange-coloured sections contain proteins appearing in both the cytoplasmic and peri/nuclear fraction, with purple lines connecting these proteins. Light orange represents proteins uniquely present in each fraction, and blue lines link different proteins belonging to the same gene ontology term; (**F**,**G**) biological functions and pathways enriched in cytoplasmic and peri-/nuclear proteomes.

In our model system we used T98G cells, in which ponatinib (PONA) inhibited the activity of PDGFR, detectable by reduced receptor autophosphorylation (Figure 1A). Importantly, ponatinib also reduced the self-renewing capacity in T98G neurospheres, which are enriched in GSCs (Figure 1B). For the detailed analysis of the adaptive response of T98G neurosphere cells to treatment with ponatinib, we used a proteomic approach.

Each treatment group was separated into two fractions: a soluble fraction, enriched in cytoplasmic proteins (C fraction) and characterised by expression of glyceraldehyde 3-phosphate dehydrogenase (GAPDH), and an insoluble fraction enriched in perinuclear and nuclear proteins (PN fraction) as indicated by histone H3 (H3) expression (Supplementary Figure S1A). Equal amounts of protein for each fraction and treatment group were precipitated, digested, and analysed by mass spectrometry. Only proteins identified by at least two peptides in both treatment groups, DMSO and ponatinib were considered for further analyses. Applying this restriction, we determined 2408 proteins in each treatment group of the C fractions and 2840 proteins in the PN fractions (Figure 1C,D). For further analysis of differential expression between treatments, we used a cut-off for a fold change (FC) of 1.3 and statistical significance of *p* < 0.05 (dotted lines in Figure 1C,D). Using these criteria, we identified a total of 776 cytoplasmic (445 up; 331 down) proteins and 395 peri/nuclear (237 up; 158 down) proteins that showed significant differential expression in response to ponatinib (Figure 1C,D). Of these, only 112 proteins exhibited ponatinib-induced changes in both, the cytoplasmic and peri/nuclear fractions with 63 of those displaying opposite changes in each fraction.

Interrogating the Reactome database for all proteins differentially expressed between peri/nuclear and cytoplasmic fractions (FC > 1.3; *p* < 0.05) using Metascape [22] confirmed an overlap of identical proteins, and also revealed that distinct proteins within each fraction contributed to the same pathways (Figure 1E). Unsupervised clustering of the Reactome pathway data identified three groups: pathways only enriched in the C fraction, pathways only enriched in the PN fraction, or pathways enriched in both fractions (Supplementary Figure S1B). In the PN fraction, protein changes were associated with chromosome maintenance, the mitotic spindle, and apoptosis execution, functions that had previously been linked to PDGFR inhibition in GBM cells [12,25]. Within the group of proteins enriched in both fractions, the most significant enrichment was in signal recognition particle (SRP)-dependent co-translation, which was accompanied by pathways linked to unfolded protein response (UPR), and rRNA modification (Supplementary Figure S1B,C). This is in line with a shift of over 30 ribosomal subunit proteins such as RPL3, 4, 6, 7, 14 or 24 and RPS10, 13 or 26 from the cytoplasm to the peri/nuclear fraction (Figure 1C). In the latter, enrichment in PERK-related activity was also detected (Supplementary Figure S1B,C), overall suggesting increased translation at the endoplasmic reticulum (ER), leading to ER stress. Intriguingly, the analysis also revealed changes in proteins related to ER-related metabolic pathways such as cholesterol biosynthesis and phospholipid metabolism in the PN fraction (Supplementary Figure S1B,C). Likewise, changes in peroxisomal import and fatty acid and lipid metabolism were detected in the PN fraction, and proteins associated with the latter pathways were also enriched in the C fraction (Supplementary Figure S1B,C). The most significant pathways that were uniquely enriched in the C fraction are linked to pyruvate metabolism, tricarboxylic acid (TCA) cycle, respiratory electron transport, and mitochondrial protein import. These changes, together with the cytoplasmic enrichment of changes in chaperonin-mediated protein folding, suggested significant alterations in the activity of mitochondria in response to ponatinib (Supplementary Figure S1B,C). Expanding our analysis to additional databases such as KEGG or GO confirmed the findings from our previous analysis, and further implied substantial changes in fatty acid and pyruvate metabolism (Figure 1F,G and Figure S1D).

### 3.2. Ponatinib Reduces Glycolysis and Enhances Lipid Metabolism and OXPHOS in T98G Neurospheres

**Figure 2:**
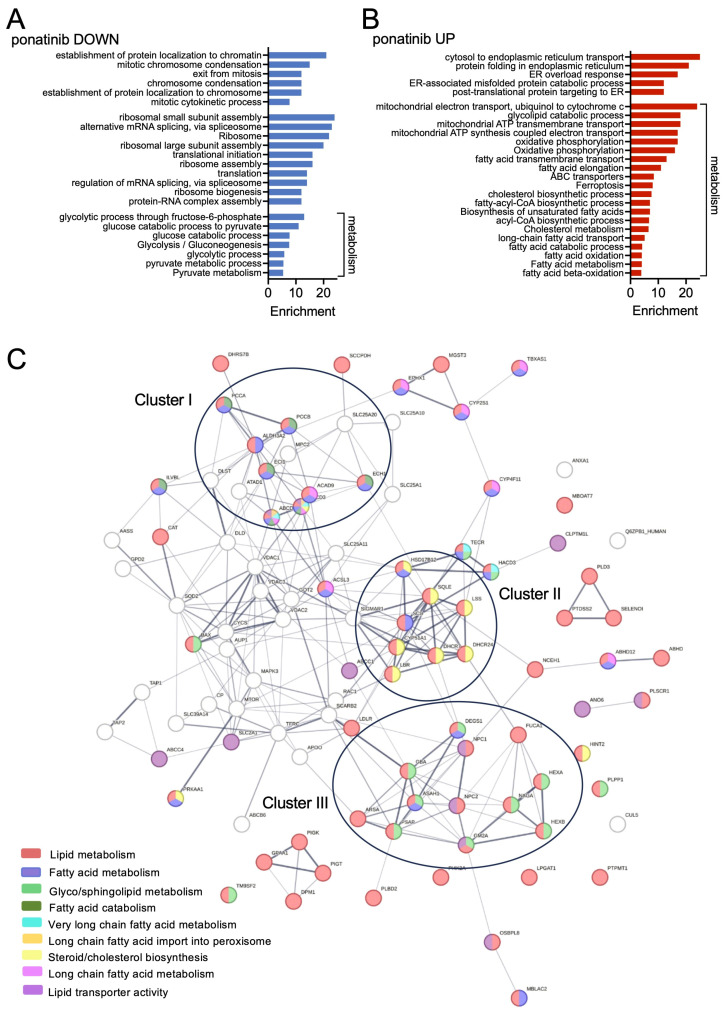
Pathway enrichment in response to ponatinib. (**A**,**B**) GO terms and KEGG pathways enriched in proteomes (**A**) down- and (**B**) up-regulated in response to ponatinib; (**C**) Network interaction analysis using STRING [21] of proteins up-regulated in T98G cells in response to ponatinib.

To better understand the cellular consequences of the observed changes, we analysed the response to ponatinib in the combined C and PN fractions with regard to up- or down-regulation. We identified 682 up- and 489 down-regulated proteins, whereby 49 proteins shared their regulatory trajectory, and 63 displayed an opposite change (Supplementary Figure S2A). Interrogation of the KEGG and GO databases detected proteins with functions linked to mitosis and chromatin organisation as well as functions and pathways linked to RNA splicing, ribosome, and translation to be down-regulated (Figure 2A and Figure S2B). On the other hand, proteins linked to translation and protein folding in the ER as well as ER overload response were up-regulated in response to ponatinib (Figure 2B and Figure S2B). Together with the results described in Figure 1, this suggests that ponatinib blocks the exit from mitosis and reduces cytoplasmic translation while enhancing translation occurring at the ER and inducing ER stress.

The ER is the primary site of lipid biosynthesis and our proteomics analysis identified functional terms and pathways linked to lipid metabolism as profoundly up-regulated in ponatinib-treated cells. Nevertheless, apart from cholesterol synthesis, which takes place in the ER, this also included ER independent pathways such as fatty acid synthesis as well as their degradation by beta-oxidation. In line with the latter, mitobiogenesis and oxidative phosphorylation (OXPHOS) were also up-regulated (Figure 2B and Figure S2B). On the other hand, we found that ponatinib down-regulated proteins linked to glycolysis and pyruvate mechanism (Figure 2A and Figure S2B).

The variety of changes in lipid metabolism we observed in ponatinib-treated neurosphere cells led us to assess the contribution of specific lipid metabolism pathways in more detail. Network interaction analysis of proteins from ponatinib-treated cells compared to control cells revealed three clusters that are all related to lipid metabolism (Figure 2C). Cluster I is represented by proteins such as ABCD1, SLC25A20, ACAD9, and ECH1 related to fatty acid transport, catabolism, and peroxisome (Figure 2C and Figure S2C). Cluster II contains proteins associated with cholesterol biosynthesis, particularly ER-located enzymes such as SQLE. Cluster III is linked to glyco/sphingolipid metabolism, whereby proteins such as HEXA, HEXB, GBA1 as well as ASAH1 are all associated with the degradation of sphingolipids and ceramide, respectively (Figure 2C).

Together, our data indicate that T98G neuropshere cells respond to ponatinib treatment with major metabolic changes by down-regulating glycolysis, up-regulating OXPHOS and generally reorganising their lipid metabolism, by up-regulating cholesterol synthesis and enhancing glyco/sphingolipid degradation as well as fatty acid catabolism. Such metabolic reorganisation in response to targeted drug treatment represents an adaption mechanism allowing for the development of resistance to therapies [26,27]. Targeting such reorganised metabolic pathways is expected to counteract the adaptation and to (re)sensitise to treatment. With this in mind, we aimed to assess the response of GSCs to treatment with ponatinib while co-targeting the rewired metabolic pathways.

### 3.3. Inhibiting Fatty Acid Beta-Oxidation Potentiates the Response to Ponatinib in GSCs

While cancer cells are generally considered to have opted for aerobic glycolysis (Warburg effect) to drive their proliferation, individual studies on the metabolic phenotype of GSCs have drawn intriguingly different conclusions. This might be due to the fact that GSCs display metabolic flexibility using both aerobic glycolysis and OXPHOS to meet their bioenergetic needs [28,29]. We had found that ponatinib-treated T98G neurosphere cells, while reducing glycolysis, increased fatty acid catabolism and OXPHOS, suggesting a greater use of and dependence on fatty acid beta-oxidation (FAO) for their energy requirements in the presence of a drug.

**Figure 3:**
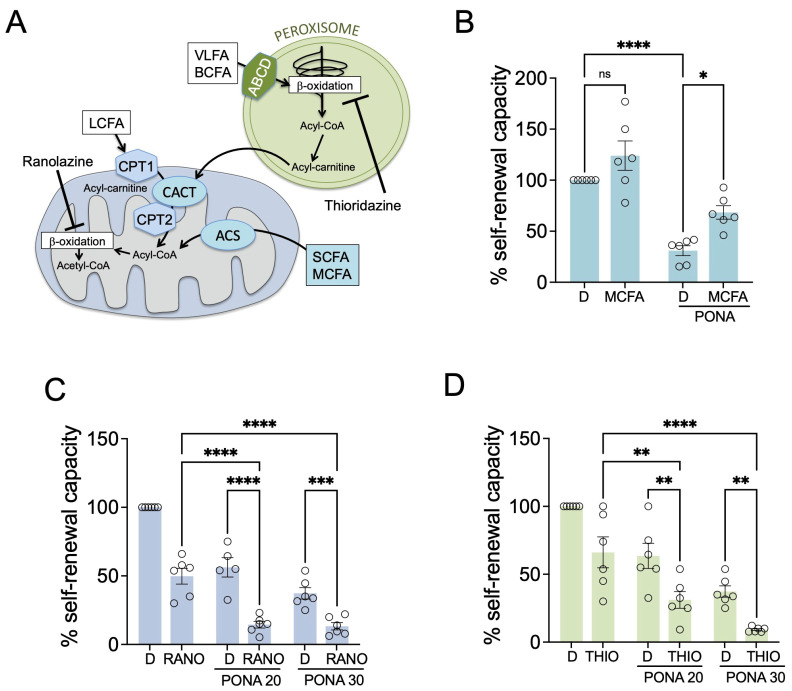
FAO inhibition modulates the response of neurosphere cells to ponatinib. (**A**) Schematic of FAO in peroxisomes and mitochondria; ABCD: ATP-binding cassette transporter; CPT1, CPT2: carnitine palmitoyltransferases; CACAT: carnitine-acylcarnitine translocase; VLFA: very-long-chain fatty acid; BCFA: branched-chain fatty acid; LCFA: long-chain fatty acid; MCFA: medium-chain fatty acid; SFA: short-chain fatty acid; ACS: acetyl-CoA synthetase; (**B**) Self-renewal analysis of T98G-neurosphere cells treated with DMSO (D), 50 nM ponatinib (PONA) and 100 µM of the MCFA decanoic acid (MCFA) as indicated; (**C**,**D**) Self-renewal analysis of T98G-neurosphere cells treated with DMSO (D) or ponatinib (20 or 30 nM) in the absence of presence of either (**C**) ranolazine (RANO, 100 µM) or (**D**) thioridazine (THIO, 1 µM). Data represent mean ± SEM. * *p* < 0.05, ** *p* < 0.01, *** *p* < 0.001, **** *p* ≤ 0.0001.

To test this hypothesis, we first assessed whether neurosphere cells use fatty acids to overcome the inhibitory effects of ponatinib. In the cell, very-long-chain fatty acids (VLFAs) enter the peroxisome through ABCD transporters (Figure 3A), and partial FAO in peroxisomes produces acyl-carnitines of short- and medium-chain fatty acids (SCFAs and MCFAs) that can enter the mitochondria through SLC25A20 (CACT). Free SCFAs and MCFAs on the other hand can enter the mitochondria by diffusion where ACS converts them into acyl-CoA, which then can be further oxidised to acetyl-CoA (Figure 3A). When decanoic acid (an MCFA) was added to the medium of untreated cells, this had a slight but not significant effect (Figure 3B). However, in ponatinib-treated T98G neurosphere cells, MCFA supplementation led to a significant alleviation (Figure 3B), indicating that fatty acid use enables these cells to counteract ponatinib-induced inhibition.

To assess the relevance of fatty acid use for ponatinib-treated GSCs, we used two different FAO inhibitors: ranolazine (RANO), an FDA-approved drug that inhibits FAO in mitochondria [30] and reduces fatty acid-driven oxygen consumption in drug-resistant melanoma cells [27], and the peroxisome-specific beta-oxidation inhibitor thioridazine (THIO) [31] (Figure 3A). Ponatinib suppressed the self-renewal capacity of T98G neurosphere cells in a dose-dependent manner, and ranolazine on its own also impacted self-renewal capacity (Figure 3C), the latter suggesting that T98G neurosphere cells under unperturbed conditions already use FAO for their propagation. Importantly, however, when these cells were treated with ponatinib in the presence of RANO this resulted in a significant further suppression of self-renewal capacity in a synergistic manner (CDI < 0.7) (Figure 3C), and a similar situation was observed with the peroxisomal FAO inhibitor THIO (Figure 3D).

**Figure 4:**
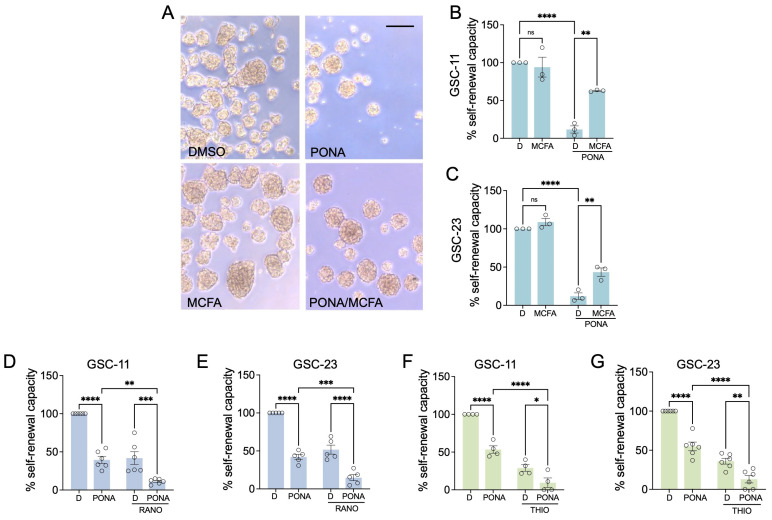
FAO inhibition modulates the response of GSCs to ponatinib. (**A**) Images of patient-derived GSC-23 glioma stem cells treated with DMSO (D), ponatinib (PONA), or decanoic acid (MCFA) either alone or in combination. Scale bar = 200 µm; (**B**,**C**) Self-renewal analysis of (**B**) GSC-11 or (**C**) GSC-23 treated as described in (**A**); (**D**,**E**) Self-renewal analysis of (**D**) GSC-11 or (**E**) GSC-23 treated with PONA in the absence of ranolazine (RANO); (**F**,**G**) Self-renewal analysis of (**F**) GSC-11 or (**G**) GSC-23 treated with PONA in the absence of thioridazine (THIO). Data represent mean ± SEM. * *p* < 0.05, ** *p* < 0.01, *** *p* < 0.001, **** *p* ≤ 0.0001.

To validate the relevance of our findings for glioblastoma stem cells we used two human patient-derived GSC cultures (GSCS-11 and GSC-23). Ponatinib effectively reduced the propagation of GSCs, but the supplementation with decanoic acid (MCFA) led to a substantial recovery from the inhibitory effect (Figure 4A–C), confirming that GSCs use fatty acids to overcome ponatinib-mediated inhibition. Furthermore, treatment with the FAO inhibitors THIO and RANO blocked the self-renewing capacity of GSC-11 and GSC23, and the combination with ponatinib significantly increased this effect in a synergistic manner (CDI < 0.7) (Figure 4D–G). This corroborates the relevance of both mitochondrial and peroxisomal FAO for GSC propagation, particularly in the presence of ponatinib.

### 3.4. Cholesterol Synthesis Protects GSCs from Ponatinib-Mediated Inhibition

**Figure 5:**
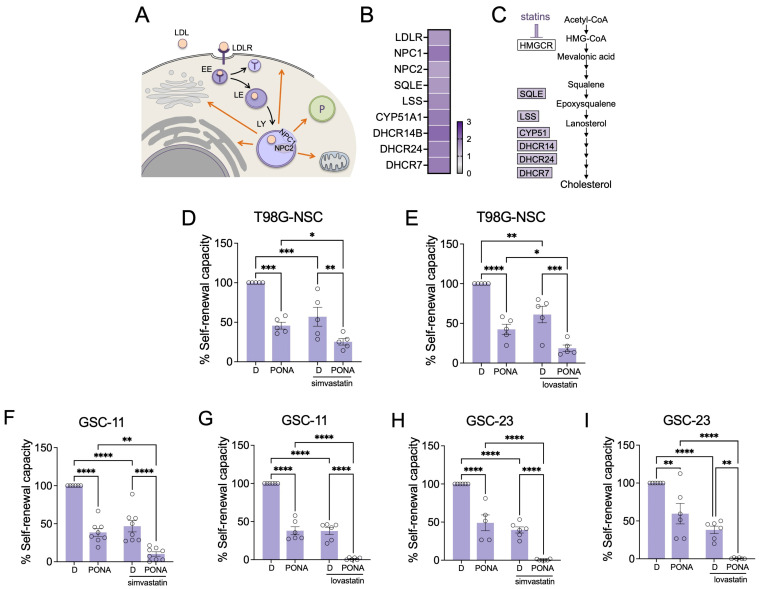
Cholesterol synthesis protects GSCs from ponatinib. (**A**) Schematic of cholesterol uptake; LDL: low-density lipoprotein; LDLR: low-density lipoprotein receptor; EE: early endosome; LE: late endosome; LY: lysosome; P: peroxisome; (**B**) Proteins up-regulated in T98G neurosphere cells in response to ponatinib; (**C**) Schematic of cholesterol synthesis. (**D**,**E**) Self-renewal analysis of T98G-neurosphere cells treated with DMSO (D) or PONA in the absence of either (**D**) simvastatin (0.2 µM) or (**E**) lovastatin (0.5 µM); (**E**–**I**) Self-renewal analysis of GSC-11 or GSC-23 treated with PONA in the absence of either simvastatin or lovastatin as indicated. Data represent mean ± SEM. * *p* < 0.05, ** *p* < 0.01, *** *p* < 0.001, **** *p* ≤ 0.0001.

Another metabolic pathway we detected to be significantly enriched upon ponatinib treatment was the cholesterol synthesis pathway (Figure 2C). Cholesterol plays a central role in the survival, growth, and migration of GBM cells [32]. Importantly, cholesterol cannot be transported across the blood–brain barrier and in a healthy brain almost all cholesterol derives from astrocytes, which synthesise cholesterol and supply it to neighbouring cells in the form of LDL [32]. GBM cells are thought to take advantage of this situation, and in EGFRvIII-over-expressing GBM cells enhancement of LDLR expression can be detected [33]. After LDLR-mediated endocytosis of cholesterol-containing LDL, free cholesterol is generated and stored in NPC1/NPC2-containing lysosomes (Figure 5A). Contact sites and fusion events between lysosomes and other organelles then mediate the cellular distribution of cholesterol [34]. GSC fitness relies on lysosomal cholesterol homeostasis, and NPC1 inhibition results in cholesterol accumulation in lysosomes leading to failure in cholesterol supply, and inducing GSC death [35].

We detected an up-regulation in LDLR, NPC1 and NPC2 in ponatinib-treated T98G neurosphere cells (Figure 5B), suggesting that these cells enhance the use of exogeneous cholesterol. Intriguingly, however, we also found that ponatinib increased the expression of various enzymes involved in endogenous cholesterol biosynthesis (Figure 5B,C). Cholesterol biosynthesis can be blocked in the initial step of the mevalonate pathway by statins, inhibitors of HMG-CoA reductase (HMGCR) (Figure 5C). Therefore, we used two different FDA-approved statins, simvastatin and lovastatin, in order to assess the relevance of cholesterol synthesis in ponatinib-treated neurosphere cells. Treatment of T98G neurosphere with simvastatin or lovastatin significantly reduced their self-renewal capacity (Figure 5D,E), but importantly, in the presence of either statin, the inhibitory effect of ponatinib was profoundly increased (Figure 5D,E). We observed an even stronger synergistic inhibitory effect of the ponatinib/statin combination (CDI < 0.7) in our two GSC cultures (Figure 5F–H), suggesting that increased intracellular cholesterol levels protect GSCs from ponatinib, and that GSCs increase cholesterol synthesis in order to counteract its inhibitory effect.

### 3.5. Ceramide Hydrolysis Counteracts Ponatinib Action in GSCs

**Figure 6:**
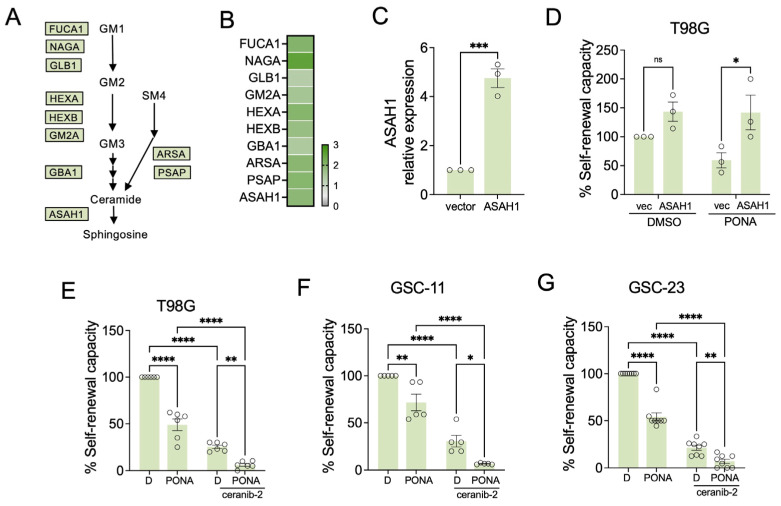
Ceramide hydrolysis counteracts ponatinib action in GSCs. (**A**) Schematic of sphingolipid salvage pathway; GM1, GM2, GM3: gangliosides; SM4: sulfatide; (**B**) Proteins up-regulated in T98G neurosphere cells as PONA response; (**C**) Expression of ectopic ASAH1 in T98G cells. (**D**) Self-renewal analysis of T98G-neurosphere cells treated with PONA with or without ASAH1 overexpression. (**E**–**G**) Self-renewal analysis of T89G, GSC-11, or GSC-23 treated with DMSO (D) or PONA in the absence of presence of ceranib-2. Data represent mean ± SEM. * *p* < 0.05, ** *p* < 0.01, *** *p* < 0.001, **** *p* ≤ 0.0001.

The third metabolic alteration we identified in ponatinib-treated GSCs was increased expression of enzymes that are part of the lysosomal sphingolipid degradation pathway [36]. The pathway involves the degradation of complex sphingolipids (e.g., glycosphingolipids, GM1 or GM2 gangliosides) to ceramides by glycosyl hydrolases (e.g., NAGA, FUCA1, GLB1, HEXA, HEXB, GBA1) and lipid-transfer proteins such as the GM2 activator protein (GM2A) and saponins (with PSAP being the precursor) (Figure 6A). We found all these central regulators of the sphingolipid salvage pathway were up-regulated in T89G neurosphere cells in response to ponatinib treatment (Figure 6B), whereas the main regulators of ceramide and sphingolipid synthesis showed no significant change.

The lysosomal sphingolipid degradation pathway converges at the production of ceramide, but in the presence of the ceramidase ASAH1, ceramide will be further hydrolysed to sphingosine [36], which can be phosphorylated by SPHK1 to form S1P [37,38]. ASAH1 is considered an important regulator of the ‘sphingolipid rheostat’, tipping the balance from pro-apoptotic ceramide to pro-survival S1P [39]. We found ASAH1 up-regulated in response to ponatinib in T98G-neurosphere cells (Figure 6B), suggesting that it could provide a survival advantage. Indeed, ASAH1 overexpression was capable of partially reversing the inhibitory effect of ponatinib on GSC self-renewal (Figure 6C,D). In contrast, ceranib-2, a ceramidase inhibitor, significantly cooperated (CDI < 0.7) with ponatinib to block self-renewal in T98G neurosphere cells (Figure 6E). Importantly, we obtained similar results in the glioblastoma stem cell cultures GSC-11 and GSC-23, where ceranib-2 suppressed self-renewal in a synergistic manner (CDI < 0.7) (Figure 6F,G).

### 3.6. Sphingolipid Degradation Is a Prognostic Factor in GBM

The metabolic pathways we identified provide a survival advantage to GSCs during the adaptation to treatment with the TKI ponatinib, and considering possible combinatorial therapies we wished to further understand whether these metabolic pathways might be of general relevance for GMB tumours. We therefore interrogated the TCGA patient cohort data [3,4] for the expression of genes we had identified in our proteomics analysis.

**Figure 7:**
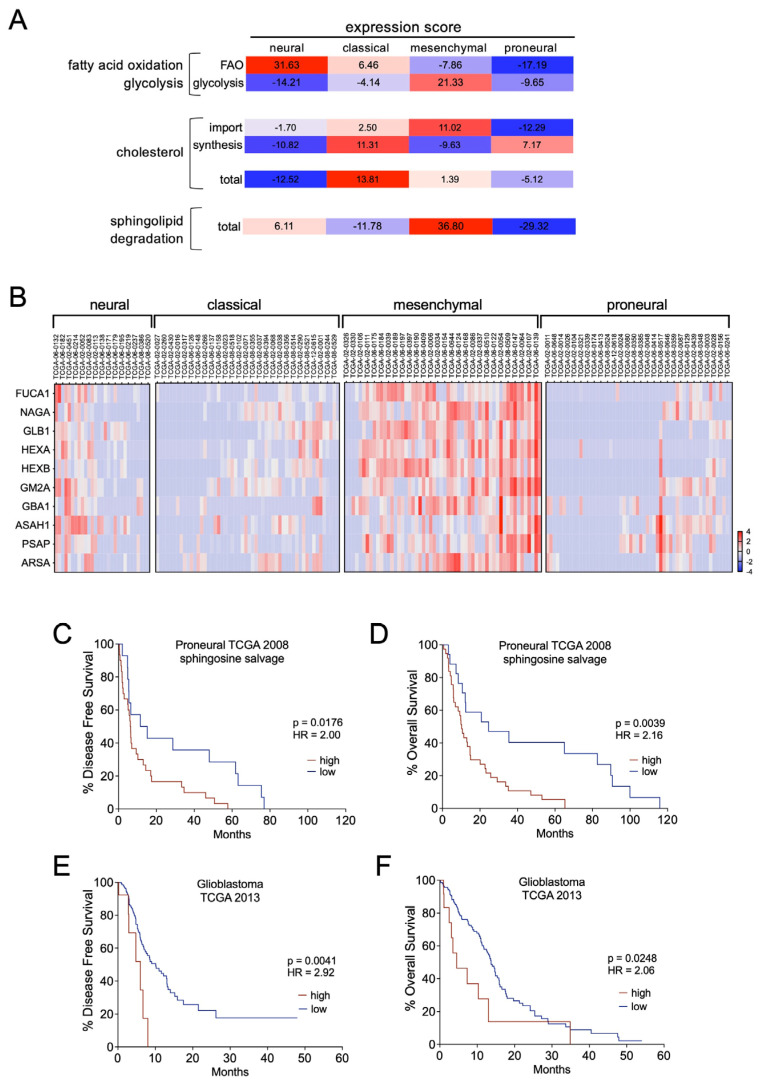
Sphingolipid degradation expression as prognostic factor in GBM. (**A**) Relative expression score of the indicated metabolic pathways considering expression data from the TCGA 2008 patient cohort [4]; (**B**) expression of the indicated genes in the indicated tumour subtypes extracted from the TCGA 2008 patient cohort [4]; (**C**,**D**) disease-free and overall survival of patients from the TCGA 2008 patient ‘proneural’ cohort [4] expressing the genes indicated in (**B**); (**E**,**F**) disease-free and overall survival of patients from the TCGA 2013 patient cohort [3], expressing the genes indicated in (**B**).

Metabolic phenotypes of GSCs have been linked to GBM subtypes, with mesenchymal GSCs displaying a high level of aerobic glycolysis [40]. Therefore, in addition to our FAO gene set we added some central glycolysis markers [27] in our analysis. In line with the findings from the corresponding GSC metabolic phenotype [40], we identified the highest expression of glycolysis markers in tumours of the mesenchymal subtype dataset (Figure 7A and Figure S3A). The two datasets corresponding to the subtypes neural and classical displayed the highest expression of our FAO gene set, whereas the proneural subtype dataset showed the lowest expression of both (Figure 7A and Figure S3A). Overall, there was great inter-tumour heterogeneity in these two energy-providing pathways even amongst the individual subtype samples, mirroring metabolic flexibility [28,29], possibly as a consequence of cells adapting to the nutrient conditions of the respective tumour microenvironments. Of note, while the link to metabolic states is so far unknown, transcriptional plasticity of individual cellular states can lead to intra-tumour heterogeneity [5].

Heterogeneity amongst TCGA subtypes was also observed, regarding cholesterol uptake and synthesis (Figure 7A and Figure S3B). The highest expression of genes linked to increased levels of intracellular cholesterol was detected in the classical subtype, whereas the lowest expression was presented in the neural phenotype. In the mesenchymal phenotype, expression of cholesterol import genes dominated over synthesis marker genes in contrast to the proneural type (Figure 7A and Figure S3B). The expression of genes linked to sphingolipid degradation resulting in ceramide hydrolysis dictated the mesenchymal phenotype with little heterogeneity (Figure 7B), suggesting a general relevance for this pathway in tumours of this subtype. Also, the neural subtype showed elevated expression and the lowest expression was found in tumours of the proneural subtype.

When we analysed the lipid metabolism regulators we identified in the adaptive response to ponatinib for their relevance in survival, FAO regulators did not show any significant correlation, and intriguingly our identified cholesterol homeostasis regulators showed opposing correlations amongst patients with mesenchymal or proneural subtype tumours (Supplementary Figure S3C,D). However, the expression of our identified sphingolipid degradation regulators displayed a significant correlation with poorer survival in patients with proneural and mesenchymal subtype tumours in the TCGA 2008 patient cohort (Figure 7C,D and Figure S3E). Moreover, they had prognostic value for disease-free as well as overall survival in the TCGA 2013 unstratified patient cohort (Figure 7E,F). Thus, amongst the three pathways we have identified, sphingolipid degradation appears to be the most general relevant pathway in GBM progression.

## 4. Discussion

Prominent genetic alterations in RTK genes in GBM have inspired the development of specific as well as multi-target TKIs [10]. However, results from clinical trials testing TKIs have been disappointing, and our knowledge about the mechanisms of resistance to TKIs in GBM is limited. Emerging evidence demonstrates that metabolic adaptation in response to drug insult is a common mechanism in therapy resistance, and we found that the reorganisation of lipid metabolism enables GSCs to counteract the inhibitory effect of the TKI ponatinib.

Our results suggest that both peroxisomal and mitochondrial FAO are involved in the adaptation of GSCs to ponatinib. These results are reminiscent of recent reports in melanoma cells where peroxisomal and mitochondrial FAO regulates the early response to BRAF inhibitors as well as the acquisition of resistance to these inhibitors [27,41,42]. While in GSC cells GBM subtypes have been linked to distinct metabolic phenotypes [40,43], in GBM tumours, we observed a more heterogeneous situation, and the balance between glycolysis and FAO marker expression varied significantly. This could be partly correlated with a GBM subtype, as for instance in ~50% of mesenchymal subtype tumours high expression of glycolysis regulators was seen. However, frequently there were tumours expressing regulators of either of the metabolic pathways or both simultaneously. This could be due to cells adapting to nutrient conditions of the respective tumour microenvironments in order to maintain their energy supply for proliferation. Distinct proliferative activity has been linked to GBM cellular states, such as astrocyte-like (AC-like) or mesenchymal-like (MES-like) and the transcriptional plasticity of these states can lead to the establishment of ‘hybrid’ states [5], which would enable individual tumours to adapt. This is entirely in line with the observation of metabolic flexibility in GSCs, which can lead to the co-existence of glycolytic and oxidative phenotypes in metabolic heterogeneous GBMs [28,29].

Our data suggest that ponatinib pushes the balance towards low glycolysis but high FAO, which is in line with the fact that PDGFRA expression inversely correlates with expression of FAO regulator genes in the TCGA patient cohort. Nevertheless, considering co-targeting FAO with RTKs in GBM in general, it should be noted that, for instance, EGFR expression positively correlates with the expression of FAO regulators, suggesting a different outcome in response to its specific inhibition.

An up-regulation of FAO, as we observe in response to ponatinib, will lead to an increase in acetyl-CoA, which can serve as substrate in the mevalonate pathway leading to cholesterol synthesis. Thus, FAO might support cholesterol synthesis by feeding acetyl-CoA to HMGCR, and similar to the inhibition of FAO, we observed synergistic effects with the HMGCR inhibitors simvastatin and lovastatin. A plethora of studies have established a role for cholesterol synthesis in the resistance to conventional chemotherapy using cisplatin, doxorubicin, or paclitaxel, and more recent evidence points to a similar function in targeted therapies [44]. Cholesterol is an integral part of cellular membranes and is most abundant in lipid rafts, membrane microdomains that contain RTKs as well as signalling proteins such as RAS GTPases [45]. Indeed, cholesterol depletion in lipid rafts disrupts PDGFR signalling [46], and an increase in cholesterol synthesis could be an adaptive response to maintain PDGFR signalling, which would explain why statins blocked the self-renewal of GSCs so potently when combined with ponatinib.

Apart from cholesterol synthesis, GBM cells can enhance LDLR expression in order to increase cholesterol uptake, and this seems to be particularly driven by EGFRvIII, and allows these cells to maintain high cholesterol levels while synthesis is suppressed [33]. We detected noticeable heterogeneity in the expression of regulators of cholesterol synthesis and uptake in tumours from patients, whereby proneural subtype tumours expressed more markers of synthesis, mesenchymal subtype tumours expressed more markers of uptake, and classical subtype tumours expressed markers of both. This heterogeneity, allowing for different means to control cholesterol homeostasis might explain why single-agent therapies using statins have failed to produce meaningful effects on GBM patients [32,47,48]. Moreover, we found the opposite correlation of cholesterol homeostasis markers with survival amongst patients with mesenchymal and proneural subtype tumours, suggesting that patient stratification should be considered when designing therapies targeting this pathway with single agents. On the other hand, our data suggest that ponatinib might drive GSCs into a dependency on cholesterol synthesis, thereby sensitising them to statins.

The third lipid metabolic pathway we found altered in ponatinib-treated GSCs is linked to the sphingolipid salvage pathway. Through degradation of complex sphingolipids, enzymes of the salvage pathway produce ceramide [36], which in the presence of ASAH1, will be converted into sphingosine, which in turn gives rise to S1P [38]. Ceramide and S1P maintain a rheostat balance and play opposing roles in apoptosis and proliferation, and in GBM tumours the sphingolipid rheostat is shifted toward S1P, resulting in a decrease in ceramides [39]. S1P has been implicated in many aspects of GBM progression including as a pro-stemness factor in GSCs [37,38,49]. Both ASAH1 and S1P are involved in therapy resistance in several cancers, including GBM [50], and we found that ASAH1 up-regulated in response to ponatinib. ASAH1 expression is also induced by ionising radiation in GBM cells and is up-regulated in radiated recurrent tumours [51], where it contributes to resistance by decreasing the apoptosis-inducing ceramide levels. ASAH1 expression is also elevated in GSCs, where its inhibition blocks proliferation [51], and several studies suggest that targeting ASAH1 using carmofur might represent a therapeutic option to target GSCs and to overcome resistance [52,53]. Nevertheless, what has not yet been considered is whether distinct GBM subtypes make different use of the sphingolipid metabolism. We found that the expression of the sphingolipid degradation regulators that were up-regulated in response to ponatinib, including ASAH1, showed high expression in ~90% of mesenchymal subtype tumours. In patients with these tumours, the highest expression was also correlated with shorter disease-free survival. Intriguingly, over 50% of tumours of the proneural subtype displayed very low expression of sphingolipid degradation regulators, yet also in this subtype, patients with high expression had shorter overall survival. This suggests that independent of subtype, increased sphingolipid degradation may further tumour progression. In line with this, we show that ponatinib up-regulates sphingolipid degradation and confers ceramidase inhibitor sensitisation in PDGFRA expressing proneural GSC-11 and GSC-23, as well as in PDGFRB expressing T98G neurosphere cells, which display mesenchymal features. Moreover, we have shown recently that dexamethasone, which is used in the majority of patients to manage the development of inflammation within the brain, particularly during treatment, up-regulates PDGFR expression and signalling in GBM cells independently of their subtype [12]. Thus, ponatinib might be applicable for a wide range of patients, and with GSCs using sphingolipid degradation to adapt to ponatinib treatment, a combination of ponatinib with ceramidase inhibition could be an attractive therapeutic approach.

## Data Availability

The original data presented in the study will be openly available from the 538 ProteomeXchange Consortium at the time of publishing.

## Supplementary Materials

The following supporting information can be downloaded at: https://www.mdpi.com/article/10.3390/pharmaceutics16060728/s1, Table S1: List of acronyms; Figure S1: Subcellular proteome analysis of ponatinib-treated T98G neutrosphere cells; Figure S2: Pathway enrichment by ponatinib in T98G neutrosphere cells; Figure S3: Metabolic pathway analysis in GBM patients.
