# Supplementary material for: Drug-Induced Reorganisation of Lipid Metabolism Limits the Therapeutic Efficacy of Ponatinib in Glioma Stem Cells
Source: Pharmaceutics. 2024 May 29;16(6):728. doi: 10.3390/pharmaceutics16060728 (PMC11206984; doi:10.3390/pharmaceutics16060728)
Supplement: Supplementary file 1 [file pharmaceutics-16-00728-s001.zip › pharmaceutics-3006566-supplementary.pdf]

Table S1. List of acronyms

ABCD	ATP binding cassette transporter D
ACS	acetyl-CoA synthetase
ALL	Acute lymphocytic leukemia
BBB	blodd brain barrier
BCFA	branched chain fatty acid
C fraction	cellular fraction
CACAT	Solute Carrier Family 25 Member 20, SLC25A20
CML	chronic myelogenous leukaemia
CPT1	carnitine palmitoyltransferase 1
CPT2	carnitine palmitoyltransferase 2
DMSO	dimethyl sulfioxide
EE	early endosome
EGFR	epidermal growth factor receptor
ER	endoplasmic reticulum
FAO	fatty acid beta-oxidation
GAPDH	glyceraldehyde 3-phosphate dehydrogenase
GBM	glioblastoma
GM1	monosialic ganglioside 1
GM2	monosialic ganglioside 2
GM3	monosialic ganglioside 3
GSC	glioblastoma
H3	histone H3
LCFA	long chain fatty acid
LDL	low-density lipoprotein
LDLR	low-density lipoprotein receptor
LE	late endosome
LY	lysosome
MCFA	medium chain fatty acid
OXPHOS	oxidative phosphorylation
P	peroxisome
PDGFR	platelet derived growth factor receptor
PN fraction	peri/nuclear fraction
PONA	ponatinib
RANO	ranolazine
RTK	receptor tyrosine kinase
SFA	short chain fatty acid
SM4	sulfatide, sulfated galactocerebroside
TCA	tricarboxylic acid
THIO	thioridazine
TKI	tyrosine kinase inhibitor
UPR	unfolded protein response
VLFA	very-long chain fatty acid

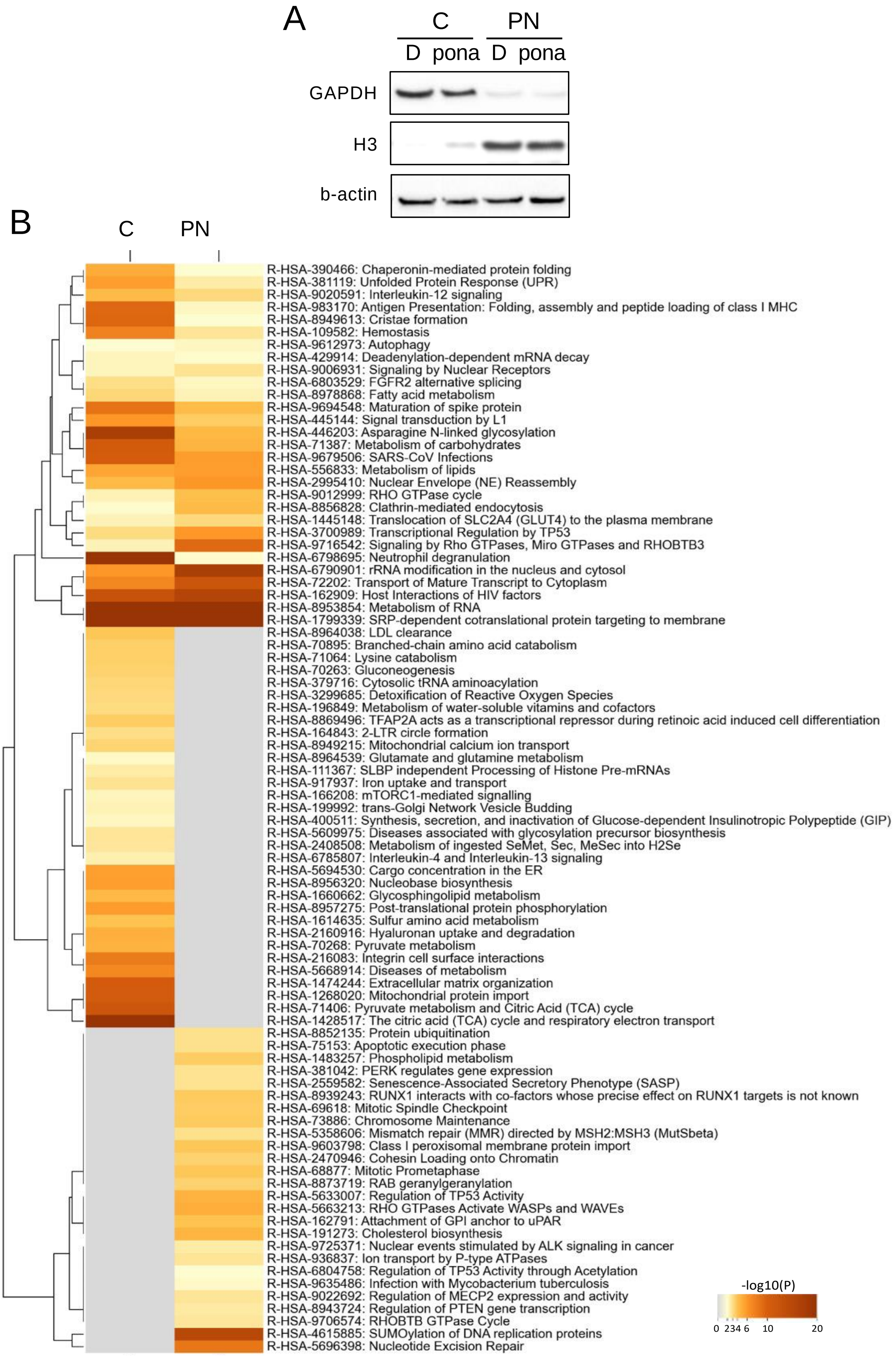
A
C
PN
D
pona
D
pona
GAPDH
H3
b-actin
B
C
PN
R-HSA-390466: Chaperonin-mediated protein folding
R-HSA-381119: Unfolded Protein Response (UPR)
R-HSA-9020591: Interleukin-12 signaling
R-HSA-983170: Antigen Presentation: Folding, assembly and peptide loading of class I MHC
R-HSA-8949613: Cristae formation
R-HSA-109582: Hemostasis
R-HSA-9612973: Autophagy
R-HSA-429914: Deadenylation-dependent mRNA decay
R-HSA-9006931: Signaling by Nuclear Receptors
R-HSA-6803529: FGFR2 alternative splicing
R-HSA-8978868: Fatty acid metabolism
R-HSA-9694548: Maturation of spike protein
R-HSA-445144: Signal transduction by L1
R-HSA-446203: Asparagine N-linked glycosylation
R-HSA-71387: Metabolism of carbohydrates
R-HSA-9679506: SARS-CoV Infections
R-HSA-556833: Metabolism of lipids
R-HSA-2995410: Nuclear Envelope (NE) Reassembly
R-HSA-9012999: RHO GTPase cycle
R-HSA-8856828: Clathrin-mediated endocytosis
R-HSA-1445148: Translocation of SLC2A4 (GLUT4) to the plasma membrane
R-HSA-3700989: Transcriptional Regulation by TP53
R-HSA-9716542: Signaling by Rho GTPases, Miro GTPases and RHOBTB3
R-HSA-6798695: Neutrophil degranulation
R-HSA-6790901: rRNA modification in the nucleus and cytosol
R-HSA-72202: Transport of Mature Transcript to Cytoplasm
R-HSA-162909: Host Interactions of HIV factors
R-HSA-8953854: Metabolism of RNA
R-HSA-1799339: SRP-dependent cotranslational protein targeting to membrane
R-HSA-8964038: LDL clearance
R-HSA-70895: Branched-chain amino acid catabolism
R-HSA-71064: Lysine catabolism
R-HSA-70263: Gluconeogenesis
R-HSA-379716: Cytosolic tRNA aminoacylation
R-HSA-3299685: Detoxification of Reactive Oxygen Species
R-HSA-196849: Metabolism of water-soluble vitamins and cofactors
R-HSA-8869496: TFAP2A acts as a transcriptional repressor during retinoic acid induced cell differentiation
R-HSA-164843: 2-LTR circle formation
R-HSA-8949215: Mitochondrial calcium ion transport
R-HSA-8964539: Glutamate and glutamine metabolism
R-HSA-111367: SLBP independent Processing of Histone Pre-mRNAs
R-HSA-917937: Iron uptake and transport
R-HSA-166208: mTORC1-mediated signalling
R-HSA-199992: trans-Golgi Network Vesicle Budding
R-HSA-400511: Synthesis, secretion, and inactivation of Glucose-dependent Insulinotropic Polypeptide (GIP)
R-HSA-5609975: Diseases associated with glycosylation precursor biosynthesis
R-HSA-2408508: Metabolism of ingested SeMet, Sec, MeSec into H2Se
R-HSA-6785807: Interleukin-4 and Interleukin-13 signaling
R-HSA-5694530: Cargo concentration in the ER
R-HSA-8956320: Nucleobase biosynthesis
R-HSA-1660662: Glycosphingolipid metabolism
R-HSA-8957275: Post-translational protein phosphorylation
R-HSA-1614635: Sulfur amino acid metabolism
R-HSA-2160916: Hyaluronan uptake and degradation
R-HSA-70268: Pyruvate metabolism
R-HSA-216083: Integrin cell surface interactions
R-HSA-5668914: Diseases of metabolism
R-HSA-1474244: Extracellular matrix organization
R-HSA-1268020: Mitochondrial protein import
R-HSA-71406: Pyruvate metabolism and Citric Acid (TCA) cycle
R-HSA-1428517: The citric acid (TCA) cycle and respiratory electron transport
R-HSA-8852135: Protein ubiquitination
R-HSA-75153: Apoptotic execution phase
R-HSA-1483257: Phospholipid metabolism
R-HSA-381042: PERK regulates gene expression
R-HSA-2559582: Senescence-Associated Secretory Phenotype (SASP)
R-HSA-8939243: RUNX1 interacts with co-factors whose precise effect on RUNX1 targets is not known
R-HSA-69618: Mitotic Spindle Checkpoint
R-HSA-73886: Chromosome Maintenance
R-HSA-5358606: Mismatch repair (MMR) directed by MSH2:MSH3 (MutSbeta)
R-HSA-9603798: Class I peroxisomal membrane protein import
R-HSA-2470946: Cohesin Loading onto Chromatin
R-HSA-68877: Mitotic Prometaphase
R-HSA-8873719: RAB geranylgeranylation
R-HSA-5633007: Regulation of TP53 Activity
R-HSA-5663213: RHO GTPases Activate WASPs and WAVEs
R-HSA-162791: Attachment of GPI anchor to uPAR
R-HSA-191273: Cholesterol biosynthesis
R-HSA-9725371: Nuclear events stimulated by ALK signaling in cancer
R-HSA-936837: Ion transport by P-type ATPases
R-HSA-6804758: Regulation of TP53 Activity through Acetylation
R-HSA-9635486: Infection with Mycobacterium tuberculosis
R-HSA-9022692: Regulation of MECP2 expression and activity
R-HSA-8943724: Regulation of PTEN gene transcription
R-HSA-9706574: RHOBTB GTPase Cycle
R-HSA-4615885: SUMOylation of DNA replication proteins
R-HSA-5696398: Nucleotide Excision Repair
-log10(P)
0 234 6 10 20

C

GO	Description	LogP	Enrichm.
R-HSA-390466	Chaperonin-mediated protein folding	-51	**48**
R-HSA-390466	Chaperonin-mediated protein folding	-21	**4**
R-HSA-381119	Unfolded Protein Response (UPR)	-58	**5**
R-HSA-381119	Unfolded Protein Response (UPR)	-27	**46**
R-HSA-6790901	rRNA modification in the nucleus and cytosol	-61	**66**
R-HSA-6790901	rRNA modification in the nucleus and cytosol	-17	**20**
R-HSA-1799339	SRP-dependent cotranslational protein targeting to membrane	-76	**22**
R-HSA-1799339	SRP-dependent cotranslational protein targeting to membrane	-35	**22**
R-HSA-381042	PERK regulates gene expression	-3	**9**
R-HSA-69618	Mitotic Spindle Checkpoint	-37	**51**
R-HSA-73886	Chromosome Maintenance	-38	**46**
R-HSA-68877	Mitotic Prometaphase	-39	**39**
R-HSA-75153	Apoptotic execution phase	-31	**69**
R-HSA-1268020	Mitochondrial protein import	-11	**91**
R-HSA-70268	Pyruvate metabolism	-48	**82**
R-HSA-1428517	The citric acid (TCA) cycle and respiratory electron transport	-49	**12**
R-HSA-71406	Pyruvate metabolism and Citric Acid (TCA) cycle	-12	**11**
R-HSA-1660662	Glycosphingolipid metabolism	-45	**63**
R-HSA-1483257	Phospholipid metabolism	-37	**37**
R-HSA-191273	Cholesterol biosynthesis	-47	**14**
R-HSA-9603798	Class I peroxisomal membrane protein import	-38	**14**

C:
PN:

D

	GO	Description	LogP	Enrichment	localisation
GO Biological Processes	GO:0006457	protein folding	-24	**74**	**C**
GO Biological Processes	GO:0050658	RNA transport	-91	**49**	**C**
GO Biological Processes	GO:0022900	electron transport chain	-27	**87**	**C**
WikiPathways	WP368	Mitochondrial long chain fatty acid beta-oxidation	-3	**86**	**C**
KEGG Pathway	hsa04216	Ferroptosis	-64	**79**	**C**
KEGG	ko00620	Pyruvate metabolism	-5	**75**	**C**
WikiPathways	WP143	Fatty acid beta-oxidation	-45	**75**	**C**
Reactome	R-HSA-159223	Mitochondrial biogenesis	-82	**61**	**C**
Reactome	R-HSA-77289	Mitochondrial Fatty Acid Beta-Oxidation	-25	**49**	**C**
KEGG Pathway	hsa01212	Fatty acid metabolism	-2	**38**	**C**
GO Biological Processes	GO:0015908	fatty acid transport	-26	**34**	**C**
GO BP	GO:0061732	mitochondrial acetyl-CoA biosynthetic process from pyruv	-56	**29**	**C**
GO Biological Processes	GO:0009062	fatty acid catabolic process	-22	**29**	**C**
KEGG	hsa_M00307	Pyruvate oxidation, pyruvate => acetyl-CoA	-37	**22**	**C**
GO BP	GO:0042776	mitochondrial ATP synthesis coupled proton transport	-92	**17**	**C**
KEGG Pathway	hsa03008	Ribosome biogenesis in eukaryotes	-10	**93**	**PN**
GO Biological Processes	GO:0050658	RNA transport	-89	**71**	**PN**
GO Biological Processes	GO:0007052	mitotic spindle organization	-59	**65**	**PN**
GO Biological Processes	GO:0007091	metaphase/anaphase transition of mitotic cell cycle	-28	**58**	**PN**
GO Biological Processes	GO:0061077	chaperone-mediated protein folding	-27	**56**	**PN**
GO Biological Processes	GO:0035337	fatty-acyl-CoA metabolic process	**-38**	**95**	**PN**
KEGG Pathway	hsa01040	Biosynthesis of unsaturated fatty acids	**-24**	**94**	**PN**
WikiPathways	WP4718	Cholesterol metabolism with Bloch and Kandutsch-Russe	**-43**	**92**	**PN**
GO Biological Processes	GO:0035336	long-chain fatty-acyl-CoA metabolic process	**-23**	**87**	**PN**
KEGG Pathway	ko00062	Fatty acid elongation	**-23**	**87**	**PN**
GO Biological Processes	GO:0000038	very long-chain fatty acid metabolic process	**-29**	**85**	**PN**
GO Biological Processes	GO:1901568	fatty acid derivative metabolic process	**-41**	**68**	**PN**

Supplementary Figure S1. Subcellular proteome analysis of ponatinib treated T98G neutrosphere cells. (**a**) Western-blot of cytoplasmic (C) and peri/nuclear (PN) fractions purified from T98-CSC cell lysates. Histone H3 (H3) and GAPDH were used as markers for the nuclear and cytoplasmic enriched fraction. Beta-actin served as loading control; (**b**) Heatmap of Reactome pathways enriched in the C and PN fractions; (**c**) Enrichment score of relevant Reactome pathways enriched in the C and PN fractions (**d**) Enrichment score of relevant gene ontology terms in the C and PN fractions.

A

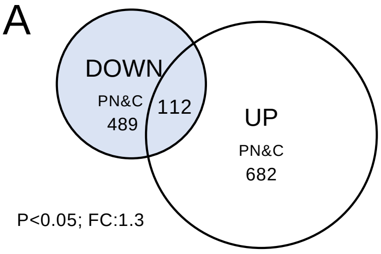

112 proteins: overlap

PN & C UP	39
PN & C DOWN	10
C UP; PN DOWN	14
C DOWN; PN UP	49

C

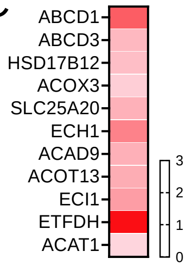

B

UP: DOWN:

Category	GO	Description	LogP	Enrichment	GeneList
GO Biological Processes	GO:0006122	mitochondrial electron transport, ubiquinol to cytochrome c	-8.2	24	UP (N+C)
GO Biological Processes	GO:0019377	glycolipid catabolic process	-6.2	18	UP (N+C)
GO Biological Processes	GO:1990544	mitochondrial ATP transmembrane transport	-3.3	18	UP (N+C)
GO Biological Processes	GO:0042775	mitochondrial ATP synthesis coupled electron transport	-31	17	UP (N+C)
GO Biological Processes	GO:0006119	oxidative phosphorylation	-42	17	UP (N+C)
KEGG Pathway	hsa00190	Oxidative phosphorylation	-41	16	UP (N+C)
GO Biological Processes	GO:1902001	fatty acid transmembrane transport	-3.8	13	UP (N+C)
GO Biological Processes	GO:0030497	fatty acid elongation	-2.6	11	UP (N+C)
KEGG Pathway	hsa02010	ABC transporters	-5.4	8.4	UP (N+C)
KEGG Pathway	hsa04216	Ferroptosis	-4.6	8	UP (N+C)
GO Biological Processes	GO:0006695	cholesterol biosynthetic process	-3.9	7.6	UP (N+C)
GO Biological Processes	GO:0046949	fatty-acyl-CoA biosynthetic process	-2.6	7	UP (N+C)
KEGG Pathway	hsa01040	Biosynthesis of unsaturated fatty acids	-2.6	7	UP (N+C)
GO Biological Processes	GO:0071616	acyl-CoA biosynthetic process	-3.6	6.7	UP (N+C)
KEGG Pathway	hsa04979	Cholesterol metabolism	-4	6.5	UP (N+C)
GO Biological Processes	GO:0015909	long-chain fatty acid transport	-3	5.1	UP (N+C)
GO Biological Processes	GO:0009062	fatty acid catabolic process	-3.2	4.2	UP (N+C)
GO Biological Processes	GO:0019395	fatty acid oxidation	-2.8	4.1	UP (N+C)
KEGG Pathway	hsa01212	Fatty acid metabolism	-2.1	4.1	UP (N+C)
GO Biological Processes	GO:0006635	fatty acid beta-oxidation	-2.1	3.9	UP (N+C)
GO Biological Processes	GO:0046967	cytosol to endoplasmic reticulum transport	-4.4	35	UP (N+C)
GO Biological Processes	GO:0034975	protein folding in endoplasmic reticulum	-5.8	21	UP (N+C)
GO Biological Processes	GO:0006983	ER overload response	-4.2	17	UP (N+C)
GO Biological Processes	GO:0071712	ER-associated misfolded protein catabolic process	-2.7	12	UP (N+C)
GO Biological Processes	GO:0006620	post-translational protein targeting to ER membrane	-3.5	12	UP (N+C)
GO Biological Processes	GO:0071169	establishment of protein localization to chromatin	-3.5	21	DOWN (N+C)
GO Biological Processes	GO:0007076	mitotic chromosome condensation	-3.9	15	DOWN (N+C)
GO Biological Processes	GO:0010458	exit from mitosis	-2.7	12	DOWN (N+C)
GO Biological Processes	GO:0030261	chromosome condensation	-6.6	12	DOWN (N+C)
GO Biological Processes	GO:1902410	mitotic cytokinetic process	-2.2	7.7	DOWN (N+C)
GO Biological Processes	GO:0070199	establishment of protein localization to chromosome	-2.7	12	DOWN (N+C)
GO Biological Processes	GO:0000028	ribosomal small subunit assembly	-8	24	DOWN (N+C)
GO Biological Processes	GO:0000380	alternative mRNA splicing, via spliceosome	-9	23	DOWN (N+C)
KEGG Pathway	hsa03010	Ribosome	-61	22	DOWN (N+C)
GO Biological Processes	GO:0000027	ribosomal large subunit assembly	-8.4	20	DOWN (N+C)
GO Biological Processes	GO:0006413	translational initiation	-15	16	DOWN (N+C)
GO Biological Processes	GO:0042255	ribosome assembly	-15	16	DOWN (N+C)
GO Biological Processes	GO:0006412	translation	-70	14	DOWN (N+C)
GO Biological Processes	GO:0048024	regulation of mRNA splicing, via spliceosome	-19	14	DOWN (N+C)
GO Biological Processes	GO:0022618	protein-RNA complex assembly	-35	12	DOWN (N+C)
GO Biological Processes	GO:0042254	ribosome biogenesis	-43	12	DOWN (N+C)
GO Biological Processes	GO:0061615	glycolytic process through fructose-6-phosphate	-3.6	13	DOWN (N+C)
GO Biological Processes	GO:0061718	glucose catabolic process to pyruvate	-2.6	11	DOWN (N+C)
GO Biological Processes	GO:0006007	glucose catabolic process	-2.2	7.7	DOWN (N+C)
KEGG Pathway	hsa00010	Glycolysis / Gluconeogenesis	-5	7.6	DOWN (N+C)
GO Biological Processes	GO:0006096	glycolytic process	-2.3	5.8	DOWN (N+C)
GO Biological Processes	GO:0006090	pyruvate metabolic process	-3.1	5.5	DOWN (N+C)
KEGG Pathway	hsa00620	Pyruvate metabolism	-2.2	5.4	DOWN (N+C)

Supplementary Figure S2. Pathway enrichment by ponatinib in T98G neutrosphere cells. (**a**) Venn diagram representing the overlap of ponatinib induced up-and down-regulated proteomes identified in cytoplasmic (C) and peri/nuclear (PN) fractions; (**b**) Enrichment score of relevant ponatinib induced up-and down-regulated gene ontology terms in the C and PN fractions. (**c**) FAO related proteins upregulated in T98G neurosphere cells in response to ponatinib.

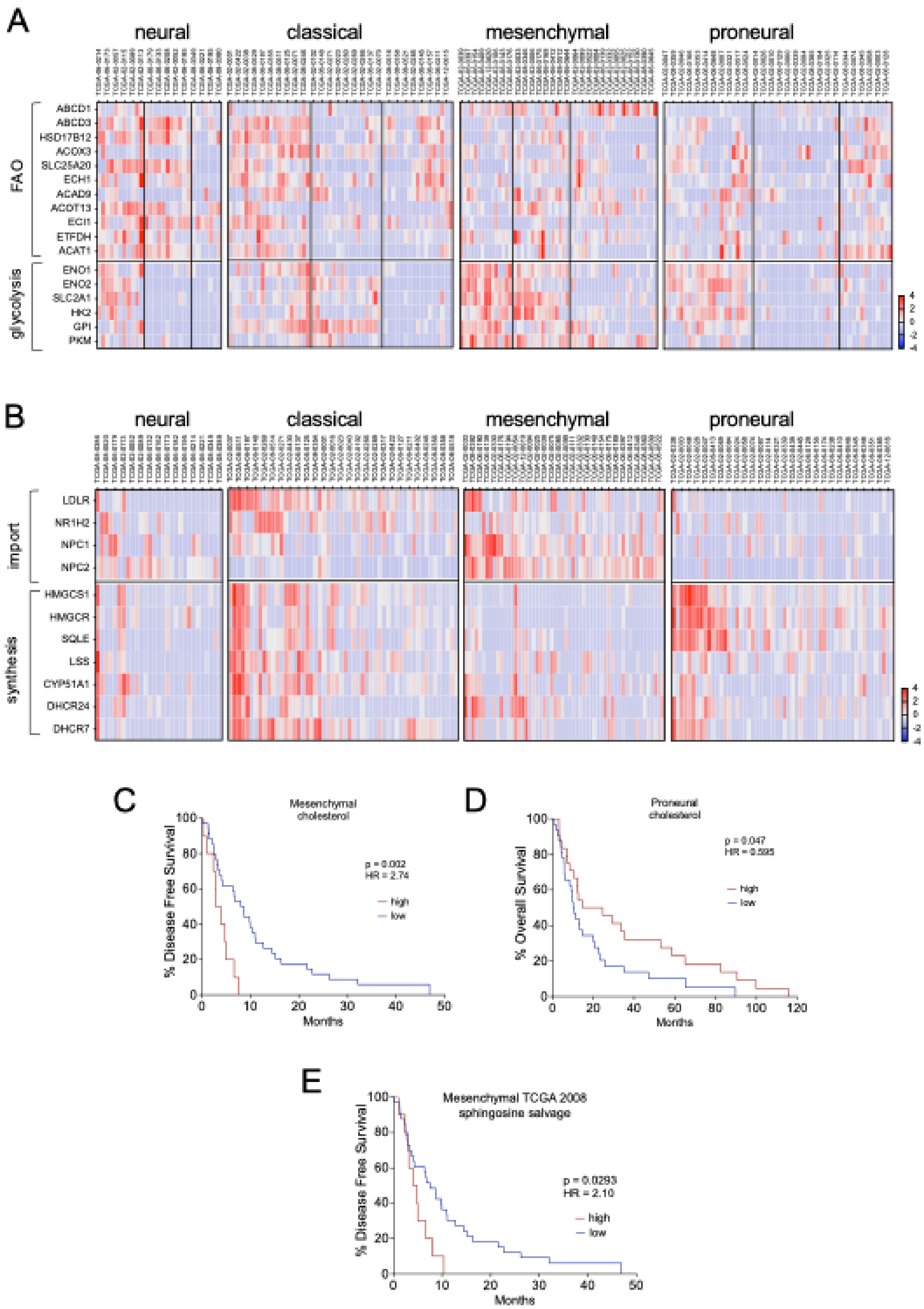

Supplementary Figure S3. Metabolic pathway analysis in GBM patients. (**a,b**) Expression of the indicated genes in the indicated tumour subtypes extracted from the TCGA 2008 patient cohort [4]; (**c-d**) Disease free and overall survival of patients from the indicated TCGA 2008 patient cohorts [4] expressing the ponatinib regulated cholesterol regulators LDLR, NPC1, NPC2, SQLE, LSS, CYP51A1, DHRC14B, DHRC24 and DHRC7. (**e**) Disease free survival of patients from the TCGA 2008 patient 'mesenchymal' cohort [4] expressing the genes indicated in Figure 7B**.**
